# On the Influence of Posture in the Treatment of Epilepsy

**Published:** 1853-07

**Authors:** Marshall Hall


From the London Lancet.

*On the Influence of Posture in the Treatment of Epilepsy.* By Dr. MARSHALL HALL.

WE have only to raise one hand and arm high above the head, and allow the other to hang down, for a minute or two, and then bring the hands together and compare the syncopal condition of the former with the apoplectic condition of the latter, to form an idea of the influence of posture in the treatment of diseases consisting of affections of the circulation, especially that of the head.

I believe ordinary syncope may pass into fatal sinking if the raised posture be continued.

I believe that simple apoplexy may become deeper and deeper, simply from the opposite course of retaining the patient in the recumbent position.

Sleep, which is a sub-apoplexy, may pass into epilepsy or apoplexy, solely from the fact of a recumbent position. As a preventive of epilepsy and apoplexy during sleep, it is of the utmost moment that the patient should habitually repose with the head and shoulders much raised. For this purpose both bed and mattress should be raised by means of a bed-chair, or triangular cushion, and the patient be prevented from sliding down in the bed by means of a firm bolster, four inches in diameter, placed under the sheet, under the front of the ischia. The trunk should be raised to an angle of 45 or 50 degrees.

In this manner the encephalon will be less oppressed with blood, the sleep will be lighter, the disposition to epilepsy or apoplexy will be diminished.

This should be the patient's habit during the rest of life.

There are two other circumstances in which attention to posture is most important.

The *first* is the condition of the patient after certain fits of epilepsy, the respiration being impeded by *rattles* in the throat. The posture should be much raised; but, besides this, it should not be such that the saliva may *fall* into the fauces. The stupor and insensibility prevent the patient from swallowing. The saliva, therefore, if a just position be not adopted, accumulates and falls into the fauces, and a throat-rattle and dyspnœa, painful to witness, and dangerous to life, are the consequences. The posture of the patient should be such as to allow the saliva to flow out of the corner of the mouth. In one case such a change of posture relieved the patient immediately.

The *second* case requiring extreme attention to the posture of the patient is that of *Syncopal Epilepsy*, or that form of epilepsy in which there is ghastly pallor of the countenance and other signs of syncopal affection. The patient should be placed with the head *low*. If this be not done, the syncope may be speedily fatal, an event which actually occurred in an interesting case a few days only ago.

The patient was no other than Ann Ross, on whom Mr. Anderson had performed the operation of tracheotomy. Her fits had changed from those of the epilepsia *laryngea* to the abortive form. The reader may remember that the patient's age was thirty-six; that her case was hereditary, her father having been epileptic; and inveterate, her fits having recurred during twenty-four years; and that she herself was thin and pallid. She was seized with syncopal epilepsy; was laid on the bed and expected to recover as formerly; was left; and was at length found to have expired. A low position and proper attention might have saved the poor creature's life.

I need scarcely observe, that what I have said of epilepsy, applies to many other diseases. It is the *principle* of position which I wish to enforce; a principle, the importance of which I believe to be still greater and still more extensive in application than is generally imagined.

